# Automated measurement and grading of knee cartilage thickness: a deep learning-based approach

**DOI:** 10.3389/fmed.2024.1337993

**Published:** 2024-02-29

**Authors:** JiangRong Guo, Pengfei Yan, Yong Qin, MeiNa Liu, Yingkai Ma, JiangQi Li, Ren Wang, Hao Luo, Songcen Lv

**Affiliations:** ^1^Department of Orthopedics and Sports Medicine, The Second Affiliated Hospital of Harbin Medical University, Harbin, Heilongjiang, China; ^2^Department of Control Science and Engineering, Harbin Institute of Technology, Harbin, Heilongjiang, China; ^3^Department of Biostatistics, School of Public Health, Harbin Medical University, Harbin, Heilongjiang, China

**Keywords:** knee, cartilage thickness, osteoarthritis, deep learning, convolutional neural network

## Abstract

**Background:**

Knee cartilage is the most crucial structure in the knee, and the reduction of cartilage thickness is a significant factor in the occurrence and development of osteoarthritis. Measuring cartilage thickness allows for a more accurate assessment of cartilage wear, but this process is relatively time-consuming. Our objectives encompass using various DL methods to segment knee cartilage from MRIs taken with different equipment and parameters, building a DL-based model for measuring and grading knee cartilage, and establishing a standardized database of knee cartilage thickness.

**Methods:**

In this retrospective study, we selected a mixed knee MRI dataset consisting of 700 cases from four datasets with varying cartilage thickness. We employed four convolutional neural networks—UNet, UNet++, ResUNet, and TransUNet—to train and segment the mixed dataset, leveraging an extensive array of labeled data for effective supervised learning. Subsequently, we measured and graded the thickness of knee cartilage in 12 regions. Finally, a standard knee cartilage thickness dataset was established using 291 cases with ages ranging from 20 to 45 years and a Kellgren–Lawrence grading of 0.

**Results:**

The validation results of network segmentation showed that TransUNet performed the best in the mixed dataset, with an overall dice similarity coefficient of 0.813 and an Intersection over Union of 0.692. The model’s mean absolute percentage error for automatic measurement and grading after segmentation was 0.831. The experiment also yielded standard knee cartilage thickness, with an average thickness of 1.98 mm for the femoral cartilage and 2.14 mm for the tibial cartilage.

**Conclusion:**

By selecting the best knee cartilage segmentation network, we built a model with a stronger generalization ability to automatically segment, measure, and grade cartilage thickness. This model can assist surgeons in more accurately and efficiently diagnosing changes in patients’ cartilage thickness.

## 1 Introduction

Knee osteoarthritis (KOA) is a common disease ranked 11th among 291 diseases in terms of causing disability across 187 countries worldwide (1). Despite 130 years of global research, the causes and pathogenesis of KOA are still not fully understood (2). KOA is a chronic disease characterized by gradual loss of cartilage, and knee cartilage declines at a faster rate with age (3). In the advanced stages of KOA, there is damage to the knee subchondral bone, degeneration of the femoral condyles and tibial plateau, and the development of osteophytes (4). For this stage, the only medical treatment available is pain management or total knee arthroplasty (5).

In general, changes in the shape of the bones around the joints usually appear 5–10 years before X-rays can capture the disease, and magnetic resonance imaging (MRI) can show abnormalities much earlier (6). Therefore, using the MRI to detect the problem and intervene in the early stages of KOA when cartilage shows insignificant wear and tear can slow down the progression of KOA (7). Measurement of knee cartilage thickness can be used to determine cartilage wear more accurately, but this is time-consuming and requires accurate measurement by an experienced imaging physician or orthopedic surgeon, which is difficult to achieve at present.

With the rapid development of artificial intelligence, deep learning (DL) has been widely used in the medical field (8), including computer-aided diagnosis, patient prognosis evaluation, and patient treatment decision-making. Convolutional neural network (CNN) (9), a key technology in DL and a sub-field of artificial intelligence, is highly effective in image recognition (10). Automatic recognition and segmentation of images through CNN have been applied to medicine, including the use of MRI images to recognize the knee meniscus and anterior cruciate ligament (11, 12). Many scholars have also studied the segmentation of knee joint cartilage in CNN (13–15), which is also the direction of our study.

In this research, our objectives are: (1) to use various DL methods to segment knee cartilage from MRIs taken with different equipment and parameters, (2) to build a DL-based model for measuring and grading knee cartilage, and (3) to establish a standardized database of knee cartilage thickness. Through this model, we hope to assist surgeons in efficiently and accurately measuring the thickness of knee cartilage and understanding changes in patients’ cartilage conditions.

## 2 Materials and methods

### 2.1 Study population

This research has been reviewed and approved by the Ethics Committee of the Second Affiliated Hospital of Harbin Medical University (Ethics Review Approval Number: KY2021-178). A total of four datasets, comprising 991 cases of MRI data, were utilized in the study.

The first dataset was retrospectively selected from a database of patients in the Second Affiliated Hospital of Harbin Medical University (hereinafter referred to as the hospital) from 2013 to 2023, totaling 516 cases. This dataset includes 225 cases for model establishment and 291 cases for building a standardized knee cartilage thickness dataset. The second dataset included 200 MRI cases obtained from the publicly available Osteoarthritis Initiative Study Protocol (OAI) dataset (16). The third dataset included 175 knee MRI cases obtained from the publicly available fastMRI dataset, and the data used in this part were obtained from the NYU fastMRI Initiative database (17). The fourth dataset consisted of 100 MRI cases obtained from SKI10 (18). All selected MRI images were in the sagittal plane, encompassing both normal-thickness cartilage and knee cartilage with altered thickness. These images were collected across multiple devices and sequences. Table 1 displays the information about the data in detail.

**Table 1:** Detailed information about the basic characteristics of the data.

	OAI	fastMRI	SKI10	Hospital
MRI scanner	Siemens Trio	Siemens Magnetom Skyra, Prisma, Biogra	GE, Siemens, Philips, Toshiba, Hitachi	Philips Achieva, NMR NeuMR
MRI sequence	3.0Tesla-IW-TSE	3.0Tesla-T2-FS	Mostly 1.5 T, some 3 T, a few 1 T,	1.5Tesla-T2-TSE
Acquisition plane	Sagittal	Sagittal	Sagittal	Sagittal
TR/TE	3200/30	4100/55; 4600/50	N/A	2900/80; 2700/70; 2600/75
Number of layers	37–39	29–36	92–120	17–19
Slice thickness (mm)	3	3;3.5	N/A	3.5;4
Columns and Row	448*444	256`1024*256`1,024	248`352*327`385	512*512; 560*560; 480*480; 528*528; 256*256
Number of subjects	200	175	100	225

“N/A” signifies that the parameter has not been provided.

### 2.2 Data labeling

For the hospital data, OAI data, and fastMRI data, annotations were labeled by two orthopedic graduate students under the supervision of experienced orthopedic surgeons (Yong Qin, 10 years of orthopedic experience; and Songcen Lv, 30 years of orthopedic experience). The annotations were performed manually in 3Dslicer (version 5.2.2). Special attention was given to the accuracy of the adjacent bones and muscle edges during the annotation process. The annotated data consisted of clear and complete cartilage images at the anterior one-fourth and posterior one-fourth positions of the sagittal plane MRI for each knee, which are crucial for assessing cartilage health.

The cartilage thickness annotation was consistently measured as the distance from the cartilage surface to the tidemark (19). The SKI10 database was segmented interactively by experts at Biomet, Inc., who segmented the femur, femoral cartilage, tibia, and tibial cartilage (18). We retained only the cartilage portions.

**Figure 1:**
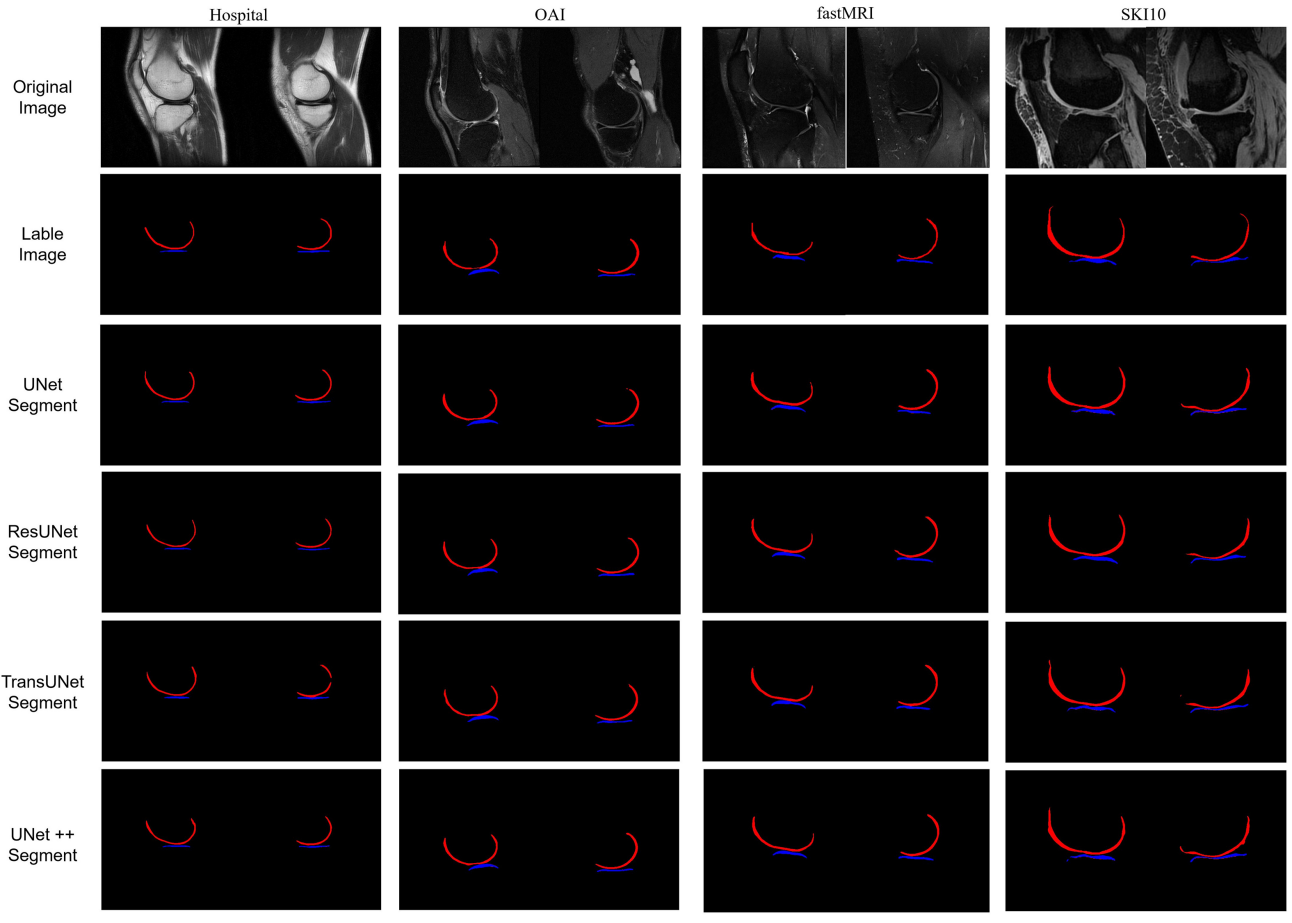
The first set represents hospital data; the second set represents OAI data; the third set represents fastMRI data; and the fourth set represents SKI10 data, which was pre-labeled. For each set, the first row displays the original MRI images of the knee joint’s medial and lateral sides. The second row shows the labeled cartilage images. The third to sixth rows depict the post-prediction images.

The final results included two labels: femoral cartilage (RGB = 255, 0, 0) and tibial cartilage (RGB = 0, 0, 255). These labels were generated in a 3D slicer, and the output images included the original knee joint MRI and the label images (Figure 1), shown in the first and second rows.

### 2.3 Cartilage classification

**Figure 2:**
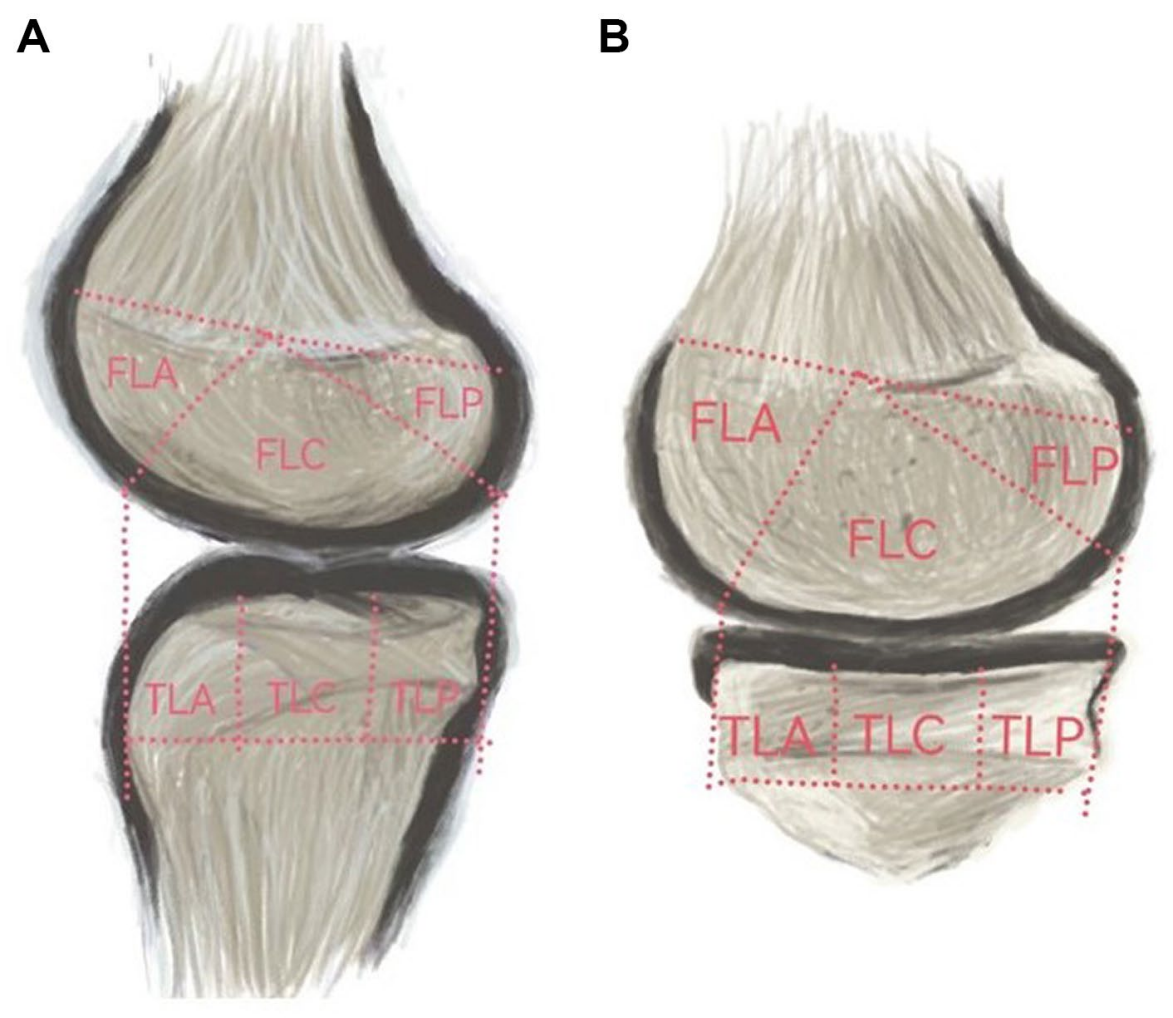
**(A)** represents the medial cartilage region, while **(B)** represents the lateral cartilage region.

Based on the classification of cartilage regions in the Whole-Organ Magnetic Resonance Imaging Score (WORMS) (20), the knee joint MRI was artificially divided into 12 regions (Figure 2): the femur region (F) and tibia region (T), the medial region (A), and the lateral region (L). Each region was further divided into three parts: anterior (A), middle (M), and posterior (C). Specifically, it includes: (1) the anterior medial femoral area (FMA); central medial femoral area (FMC); and posterior medial femoral area (FMP); (2) the anterior lateral femoral area (FLA); central lateral femoral area (FLC); and posterior lateral femoral area (FLP); (3) the anterior medial tibial area (TMA); central medial tibial area (TMC); and posterior medial tibial area (TMP); and (4) the anterior medial tibial area (TLA); central medial tibial area (TLC); and posterior medial tibial area (TLP).

### 2.4 Deep learning methods

We conducted a comparison of four deep learning segmentation models: UNet (21), UNet++ (22), ResUNet (23), and TransUNet (24). This study involved 700 samples, comprising a total of 2,800 images for network training, with the training results displayed in Figure 1, shown in the third to sixth rows. Before training, all images were resized to a uniform size (480×480 pixels) and normalized. The dataset was divided into a training set, a test set, and a validation set, in the ratio of 6:2:2. The aforementioned algorithms were implemented under the PyTorch (CUDA 11.8) framework, and computations were performed on a tower server composed of 4 NVIDIA 12GB GPUs.

UNet: The most widely used CNN in medical image processing. Its structure is U-shaped and includes symmetrical encoders and decoders. After inputting MRI images, the encoder extracts cartilage features through convolution layers and pooling operations, reducing the image size from 480*480 to 30*30. Subsequently, using Concat to connect multiple tensors, the model generates predictive results through layer-by-layer upsampling and per-pixel classification.

UNET++: Builds upon UNet by introducing dense skip connections and multi-scale feature fusion. It captures features from different levels, integrating them through feature stacking, thereby enhancing the extraction of knee joint cartilage features.

ResUNet: Based on UNet, it introduces residual connections (similar to ResNet), allowing the image to be passed not only to the next layer but also directly to deeper layers through skip connections, effectively reducing the problem of gradient vanishing during training.

**Figure 3:**
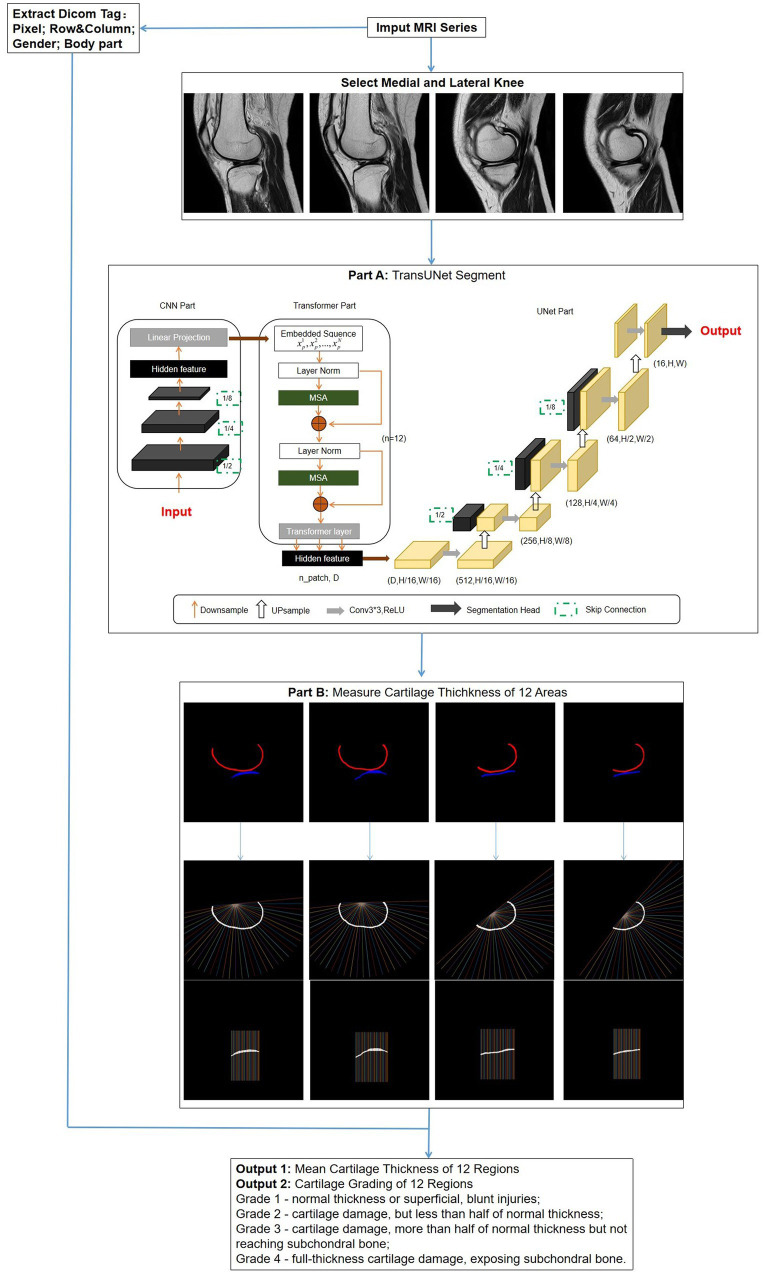
Overall flowchart of the research model. After the images are selected, a network is used to segment the regions of cartilage. Subsequently, the segmentation results are automatically measured for thickness and graded. Simultaneously, DICOM tag information is extracted for obtaining standard knee joint cartilage thickness. **Part A**: The structure of TransUNet, which achieved the best results of all network models; **Part B**: measurement method of cartilage thickness; vertical lines 1–12 intersecting with cartilage represent FMA and FLA; lines 13–24 represent FMC and FLC; and lines 25–36 represent FMP and FLP.

TransUNet: This is a segmentation network based on the transformer model, using a hybrid architecture of CNN-Transformer-UNet. Initially, it employs the feature encoding part of CNN to extract features while reducing the image size from 480*480 to 30*30, followed by extracting global contextual information using the self-attention mechanism of the transformer. Finally, the UNet decoder upsamples the encoded features, which are combined with different resolution CNN features extracted from the encoder path, achieving precise localization (Figure 3, Part A).

**Figure 4:**
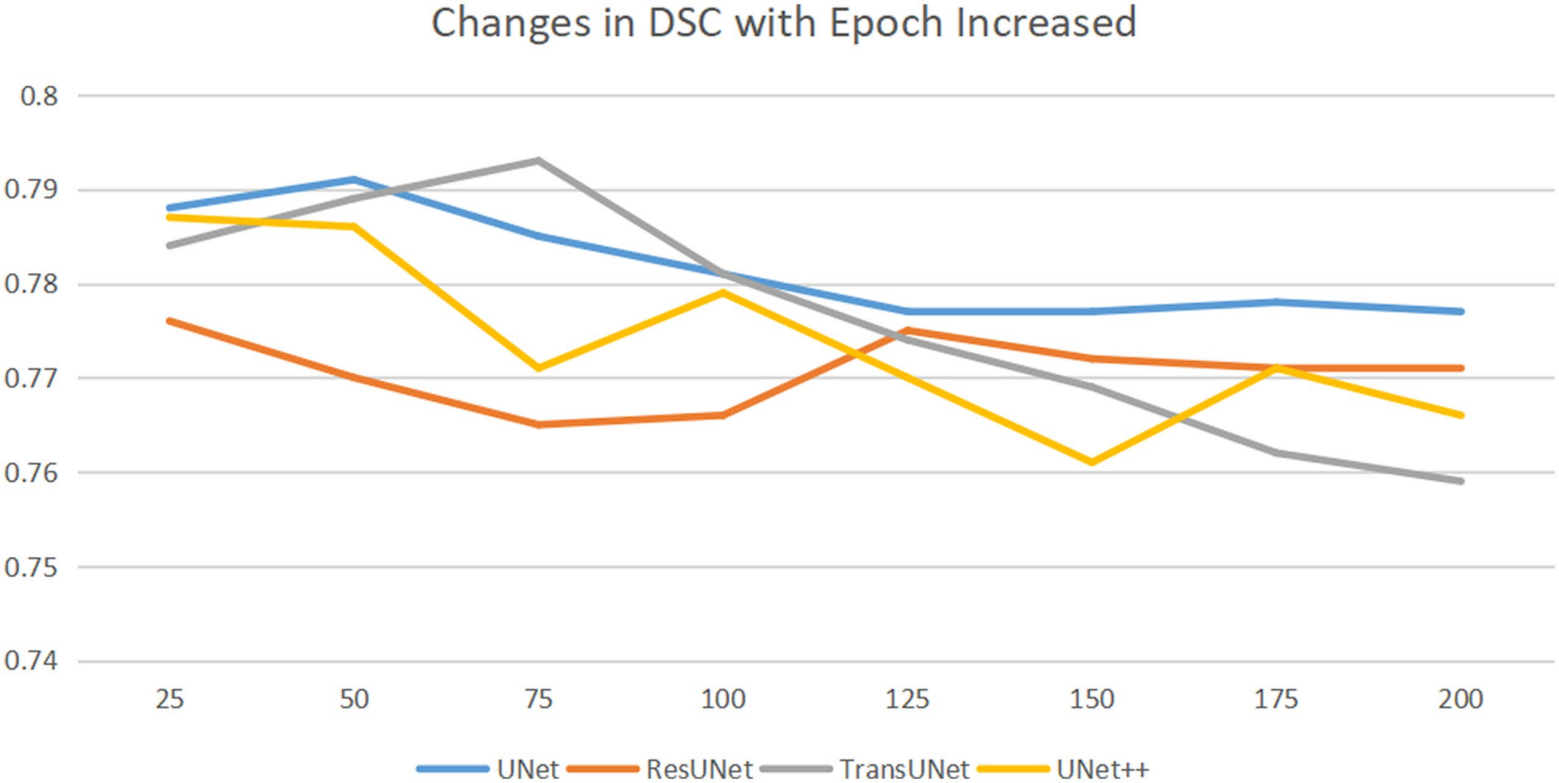
Horizontal axis is epoch, and the vertical axis is DSC. DSC changes as the epoch increases. As the epochs increase, the optimal DSC occurs at different epochs for different networks. We saved the network weights corresponding to the best DSC.

To train the network, the gradient descent method was used (25), with a batch size of 16, a weight decay of 1e-4, and a learning rate drop factor of 0.1. The initial learning rate was 2e-4. After every 100 training sessions, the learning rate = initial learning rate * drop factor until convergence. We used cross-validation method to explore the optimal epochs, with epochs selected from 50, 75, 100, 125, 150, 175, 200, specific epochs for each type of network are shown in Figure 4.

### 2.5 Cartilage thickness measurement

Thickness measurement is implemented in MATLAB (R2022a). First, the post-segmentation data are processed to remove small independent pixels. Then, the cartilage range was determined by identifying the pixel points corresponding to different colors of femoral and tibial cartilage. The thickness calculation was an improvement on traditional methods (26), expanding the detection area for increased accuracy. For the femoral side, the midpoint of the line connecting the endpoints on both sides was taken as the origin; from this point, 36 vertical lines intersecting the cartilage were drawn at 5° intervals. Similarly, the tibial cartilage was divided into 36 vertical lines by taking the sides as endpoints (Figure 3, Part B).

The number of pixel points obtained from the intersections was calculated based on the Digital Imaging and Communications in Medicine (DICOM) tag information of the original MRI, including row and column, as well as the actual distance represented by each pixel (pixel spacing). This calculation provided the cartilage thickness at each point. The average thickness for each region was computed separately for lines 1–12, 13–24, and 25–36.

The thickness measurement results were classified into four levels according to Recht grading (27) and ICRS grading (28): Grade 1 – normal thickness or superficial, blunt injuries; Grade 2 – cartilage damage, but less than half of normal thickness; Grade 3 – cartilage damage, more than half of normal thickness but not reaching subchondral bone; Grade 4 – full-thickness cartilage damage, exposing subchondral bone. By extracting gender and body part information (left or right knee) from the DICOM tags and using the corresponding standard cartilage thickness, the model determines the grading. Ultimately, this model provides surgeons with displays of the cartilage thickness and grading for each region of the knee joint (Figure 3).

### 2.6 Selection of normal knee cartilage

**Figure 5:**
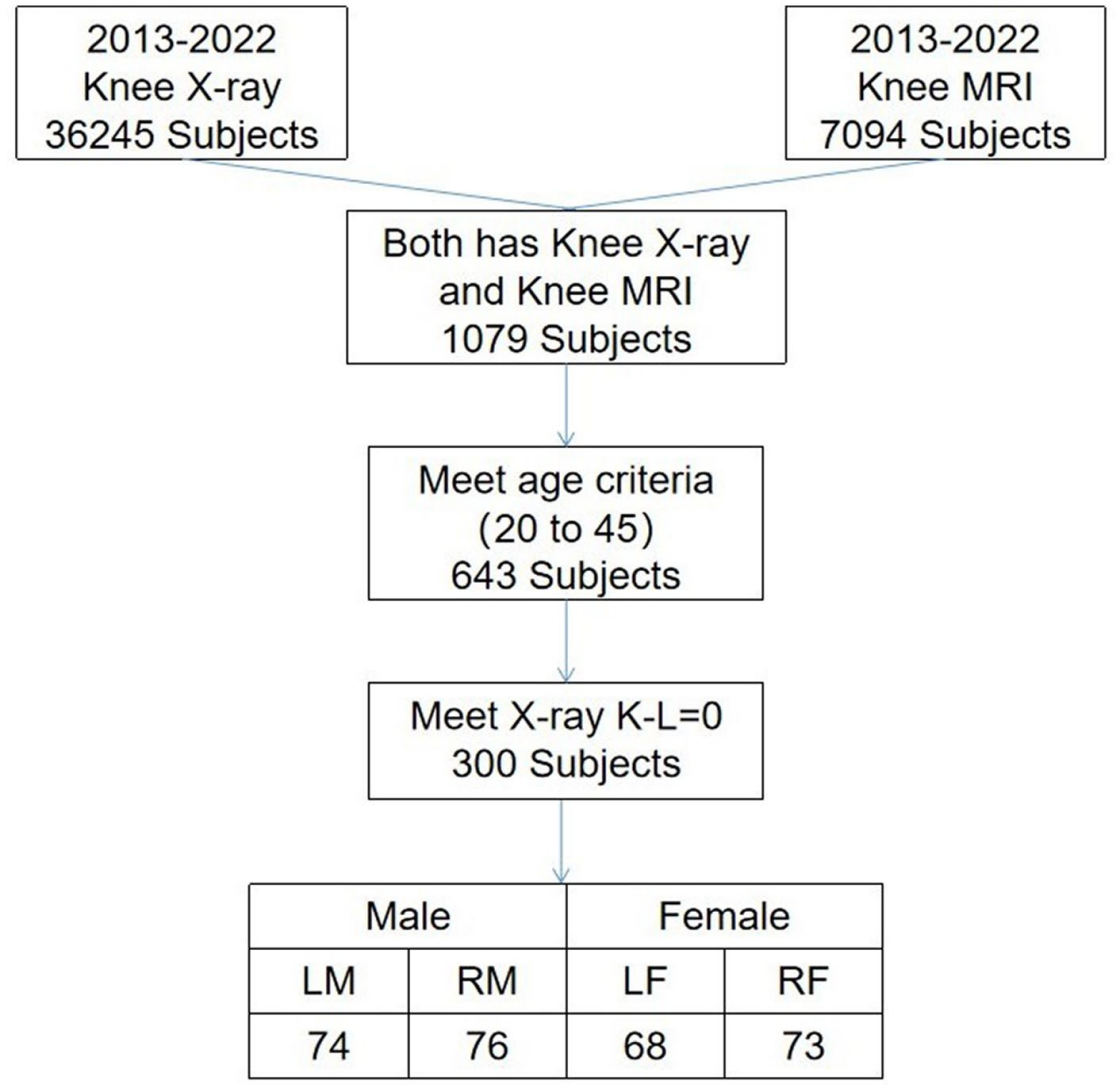
Selection criteria and the number of stranded cartilages. Man’s left knee (LM), man’s right knee (RM), woman’s left knee (LW), and woman’s right knee (RW).

From a pool of 7,094 cases spanning 2013–2022, we screened 291 knee joint MRI cases defined as having normal cartilage thickness. These cases were considered the standard for knee cartilage thickness in the northeastern region of China. Standard knee cartilage thickness was defined as individuals aged 20–45 with X-ray Kellgren–Lawrence gradings of 0 or 1. All these MRI images were sourced from the hospital dataset. Some individuals may be entirely normal, while others may have accompanying conditions such as meniscal injuries or bone abnormalities; however, their cartilage remains intact. We defined standard cartilage thickness as aged 20–45, as cartilage below 20 years of age is in constant growth and change, and cartilage thickness tends to wear to varying degrees with increasing age beyond 45 (29). The specific screening results are presented in Figure 5.

### 2.7 Statistical analysis

In this study, the collected data were statistically analyzed using MATLAB. The accuracy of cartilage segmentation was evaluated using the dice similarity coefficient (DSC) and Intersection over Union (IoU). Both DSC and IoU values ranged from 0 to 1, with higher values indicating better segmentation performance. The accuracy of cartilage thickness measurement was assessed using the mean absolute percentage error (MAPE). A MAPE of 0% indicates a perfect model, while a MAPE greater than 100% suggests a poor-quality model. The grading accuracy of the model was validated using the metric ‘Accuracy’. The standard cartilage thickness results inferred were presented as mean ± standard deviation (¯x ± s) for measurements following a normal or near-normal distribution.

## 3 Results

### 3.1 Segmentation results in different CNNs

**Figure 6:**
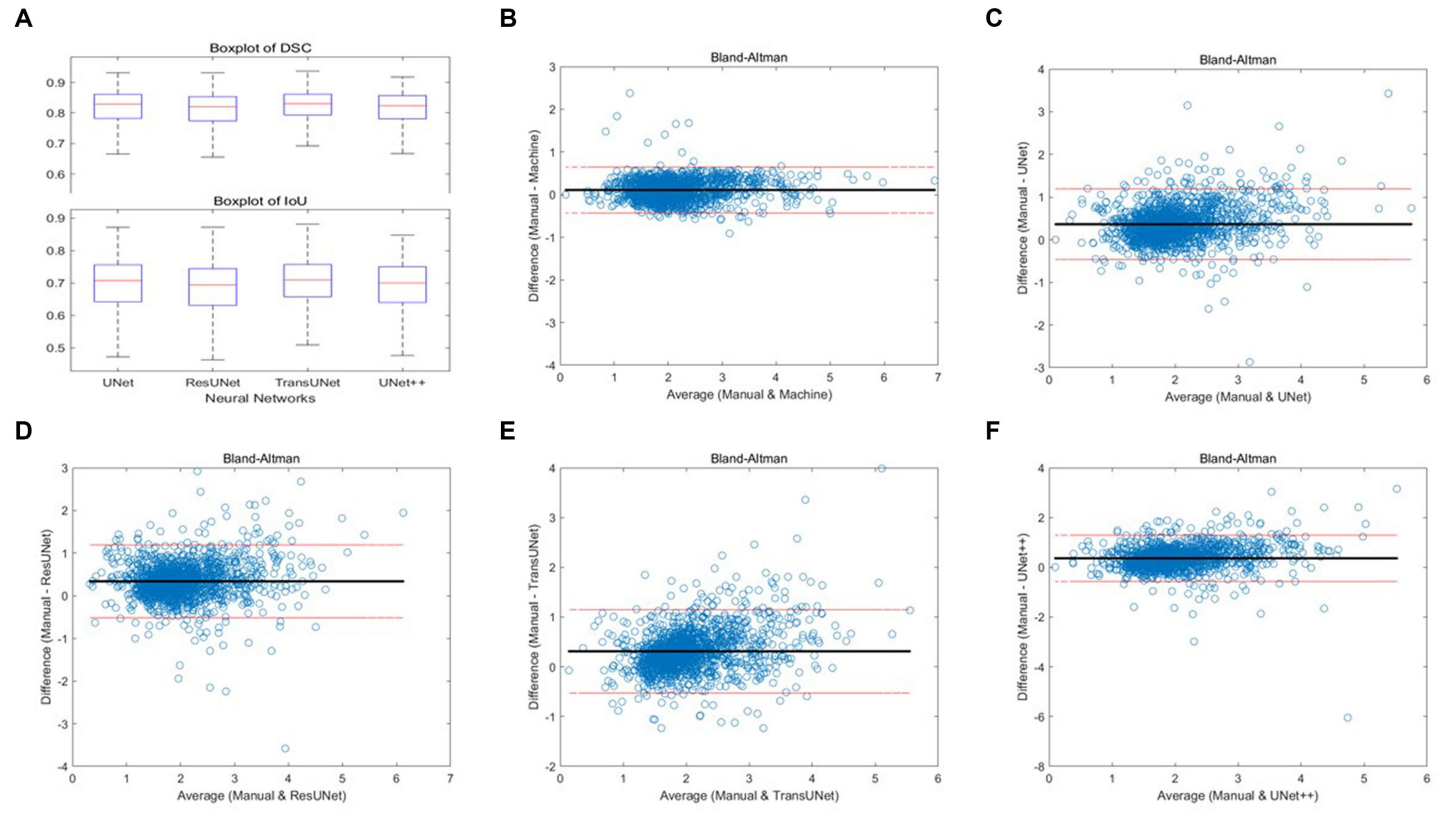
**(A)** Boxplots of total DSC and IoU for four different networks; **(B–F)** Bland–Altman consistency analysis of cartilage thickness measurement results under different methods.

We employed four CNN models for segmentation training on the mixed dataset. The results indicate that the TransUNet model achieved the best segmentation performance, with a DSC of 0.823 for segmenting femoral cartilage, 0.803 for segmenting tibial cartilage, 0.813 for overall DSC, and 0.692 for overall IoU. Other network models’ performances were less impressive than TransUNet’s, particularly in tibial cartilage segmentation, with DSCs ranging from 0.776 to 0.788. The specific segmentation results for each network are shown in Table 2 and Figure 6, Part A.

**Table 2:** Result in different CNNs.

	DSC			IoU		
	Femur	Tibia	Total	Femur	Tibia	Total
UNet	0.834	0.776	0.805	0.720	0.642	0.681
UNet++	0.822	0.780	0.801	0.704	0.650	0.677
ResUNet	0.800	0.788	0.794	0.674	0.665	0.669
TransUNet	0.823	0.803	0.813	0.704	0.680	0.692

DSC: dice similarity coefficient; IoU: Intersection over Union.

### 3.2 Validation of automatic cartilage thickness measurement accuracy

Two orthopedic graduate students manually measured and graded the cartilage thickness in 12 regions of 135 knee MRI samples from the test set under the supervision of experienced orthopedic surgeons and compared the results with the results from the automatic thickness measurement and grading model, showing the overall MAPE of the thickness measurement was 10.10% and the grading accuracy was 89.99%. Compared with the results of automatic measurement after different networks’ segmentation, the overall MAPE of thickness measurement ranged from 17.47 to 20.34%, and the grading accuracy ranged from 75.79 to 83.08%, with TransUNet showing the best performance. The specific segmentation results are shown in Table 3 and Figure 6, Part B–F.

**Table 3:** MAPE between manual measurements and grading and automatic measurements and grading, as well as between manual measurements and grading and measurements and grading from different networks segmentation, *p* < 0.05.

MAPE of thickness measurement					
	Automatic	UNet	UNet++	ResUNet	TransUNet
Medial Femur	6.06%	15.18%	18.96%	20.15%	15.46%
Lateral Femur	7.70%	12.18%	15.97%	18.14%	15.02%
Medial Tibia	13.38%	25.53%	24.54%	22.34%	20.68%
Lateral Tibia	13.26%	23.45%	21.89%	18.73%	18.73%
Total	10.10%	19.09%	20.34%	19.84%	17.47%

Accuracy of grading					
	Automatic	UNet	UNet++	ResUNet	TransUNet
Medial Femur	93.08%	86.17%	76.79%	80.74%	78.02%
Lateral Femur	90.12%	90.86%	76.29%	81.97%	82.96%
Medial Tibia	87.40%	60.49%	66.66%	76.04%	84.19%
Lateral Tibia	89.38%	71.35%	82.22%	89.13%	87.16%
Total	89.99%	77.21%	75.49%	81.97%	83.08%

For a unified statistical analysis, we aggregated the data from the 12 regions into four areas.

### 3.3 Normal knee cartilage thickness

The results of the cartilage thickness analysis of 291 normal knee cartilage thicknesses are shown in Table 4. The results showed that the cartilage thickness of normal knee joints ranged from 1.79 ± 0.26 mm to 3.13 ± 0.54 mm. In general, the average thickness of femoral cartilage is 1.98 mm, and the average thickness of tibial soft tissue is 2.14 mm. The distribution of the cartilage thickness was uneven, with the medial femur being thicker than the lateral femur, the tibia cartilage thickness being greater than that of the femur, and the thickness being greatest in the central medial aspect of the tibia. Judging by gender and considering both right and left knees, the cartilage thickness was generally greater in men than in women,” and the cartilage thickness of the right knee was generally greater than the left knee.

**Table 4:** Standard thickness of cartilage (x ± s), in mm.

Femur						
	FMA	FMC	FMP	FLA	FLC	FLP
LM	2.14 ± 0.29	1.97 ± 0.21	2.02 ± 0.32	1.98 ± 0.19	2 ± 0.27	1.89 ± 0.38
LW	1.95 ± 0.35	1.92 ± 0.23	1.95 ± 0.23	1.97 ± 0.18	1.88 ± 0.21	1.92 ± 0.14
RM	1.98 ± 0.24	2 ± 0.23	2.06 ± 0.42	2.3 ± 0.22	2.06 ± 0.27	1.98 ± 0.37
RW	1.91 ± 0.21	1.88 ± 0.2	1.89 ± 0.22	2.01 ± 0.21	1.93 ± 0.18	1.87 ± 0.39

Tibia						
	TMA	TMC	TMP	TLA	TLC	TLP
LM	2.13 ± 0.32	2.98 ± 0.58	2.34 ± 0.54	1.86 ± 0.28	2.02 ± 0.39	1.82 ± 0.23
LW	2.07 ± 0.32	2.74 ± 0.6	2.14 ± 0.39	1.72 ± 0.21	1.83 ± 0.36	1.79 ± 0.26
RM	2.18 ± 0.31	3.13 ± 0.54	2.43 ± 0.47	1.88 ± 0.3	2.14 ± 0.5	1.91 ± 0.28
RW	2 ± 0.3	2.71 ± 0.42	2.26 ± 0.38	1.68 ± 0.2	1.88 ± 0.35	1.76 ± 0.21

## 4 Discussion

In this study, we utilized various CNNs to train, segment, measure, and grade the knee cartilage thickness in 12 regions of a mixed knee cartilage dataset. We developed a measurement and grading model under DL that is applicable to different sequences. Currently, assessing changes in knee cartilage thickness requires a significant amount of effort and time through a manual MRI examination. With limited time for diagnosis and treatment, surgeons may struggle to focus on each patient’s cartilage thickness, making early detection of mild knee joint cartilage wear challenging. This study primarily focuses on the efficient and accurate measurement of cartilage thickness, aiming to assist surgeons in understanding changes in patients’ cartilage.

In previous studies, scholars such as Norman (29) utilized the OAI dataset for segmentation in UNet, achieving a DSC of 0.770 to 0.878. Additionally, another study (30) employed SegNet for segmentation in a mixed dataset combining SKI10 and personal data, obtaining a volumetric overlap error (VOE, the opposite of DSC) coefficient of 0.289, higher than the UNet’s 0.351. Currently, many researchers (30–32) prefer using individual datasets for segmentation, yielding DSC coefficients typically ranging between 0.74 and 0.87. Therefore, uncertainties exist regarding the reliability of these models when applied to MRI scans from different devices or sequences. We attempted to use the independent hospital dataset for training under UNet and obtained results of femur DSC of 0.86, tibia DSC of 0.79, and overall DSC of 0.82. However, when the network trained using the hospital dataset was applied to the OAI or fastMRI datasets, the DSC was only 0.47 to 0.53, which suggests that models trained on individual datasets exhibit subpar performance when inferring on MRI scans acquired with other devices and sequences. To enhance the effectiveness and robustness of the thickness measurement model, we opted for training on a mixed dataset. While a mixed dataset may influence the recognition accuracy and decrease precision to some extent, the model still demonstrates favorable performance across different MRI devices and sequences.

We employed four different network models for knee cartilage segmentation. UNet is the most widely used DL model in knee joint segmentation currently; however, other networks, traditionally applied to the segmentation of organs such as the lungs, heart, stomach, and brain (33–36), are now being explored in this area. This study is the first to apply these network models to knee cartilage segmentation, with results indicating that some networks outperform UNet in terms of segmentation accuracy. The results demonstrate that the best-performing network currently is TransUNet. Compared to other networks, TransUNet more effectively captures long-distance dependencies and global contextual information within images, accurately recognizes and differentiates varying degrees of cartilage damage, and shows superior capabilities in processing edge pixel details.

Measuring cartilage thickness manually is a time-consuming task. Shepherd and Seedhom (37) first measured 11 cadavers in 1999, revealing an average cartilage thickness of 2.15 mm on the surface of the femur, 2.01 mm in the tibial plateau covered by the meniscus, and 2.59 mm in the uncovered region of the tibia. Cohen et al. (38) proved that cartilage thickness measured via MRI is basically the same as direct measurements, with an average femoral cartilage thickness of 2.08 mm and an average tibial cartilage thickness of 2.32 mm. Our results also show a similar trend, with an average cartilage thickness of 1.99 mm in women and 2.13 mm in men.

In recent years, some researchers have already utilized DL methods to measure cartilage. Shah (31) first employed UNet for the segmentation and measurement of normal knee cartilage thickness, demonstrating that DL can effectively measure thickness on MRI. Si (32) also utilized UNet for segmentation, measuring cartilage thickness through a dot-product approach. Liu (30) and other researchers used the Eulerian PDE (39) approach in V-Net for measurement. In contrast, our approach involves pixel-level recognition and calculation, building a model capable of measuring the thickness of cartilage automatically after segmentation and validating its reliability. In order to make the results more applicable to clinical practice, we graded the measurement results to help surgeons effectively assess changes in cartilage thickness in patients and provide valuable reference for subsequent treatment decisions.

There are some limitations to this study. First, the lack of comparison with other scholars’ cartilage thickness measurement methods limits our ability to conclusively ascertain the distinct advantages or enhancements that our method offers over existing methods. Second, as our normal knee cartilage thickness data only represents the average thickness level in Northeast China, it may not be representative of other regions in China or other countries and races. Additionally, our cartilage thickness standards are limited to gender and body part; we have not considered other patient factors such as height, age, and BMI. It is unknown whether these factors are related to standard cartilage thickness; therefore, it is necessary to continue to collect data and establish a more complete standard knee cartilage thickness dataset. Finally, our scoring system is a retrospective study and provides a universally applicable thickness measurement score primarily for assessing early cartilage damage. It serves as an auxiliary tool for surgeons and may require individual judgments in certain special cases.

## 5 Conclusion

In conclusion, we selected the best knee cartilage segmentation network and built a model of automatic knee cartilage segmentation, measurement, and grading. Through this model, the effectiveness and robustness of processing images obtained under different MRI devices and parameters have been enhanced. It can help surgeons more accurately and efficiently diagnose changes in cartilage thickness in patients. At the same time, we have defined the standard cartilage thickness in northeast China. We hope that after further research and the collection of large amounts of data, we can build a global dataset of standard knee cartilage thickness to help more patients and surgeons.

## Data availability statement

The raw data supporting the conclusions of this article will be made available by the authors, without undue reservation.

## Ethics statement

Written informed consent was obtained from the individual(s) for the publication of any potentially identifiable images or data included in this article.

## Author contributions

JG: Writing – original draft, Writing – review & editing. PY: Methodology, Writing – review & editing. YQ: Resources, Supervision, Writing – original draft. ML: Writing – review & editing, Supervision, Funding acquisition. YM: Writing – original draft. JL: Methodology, Software, Writing – review & editing. RW: Data curation, Formal analysis, Writing – review & editing. HL: Funding acquisition, Supervision, Writing – review & editing. SL: Funding acquisition, Supervision, Writing – review & editing.
